# Identification of putative markers linked to grain plumpness in rice (*Oryza sativa* L.) via association mapping

**DOI:** 10.1186/s12863-017-0559-6

**Published:** 2017-10-12

**Authors:** Erbao Liu, Siyuan Zeng, Xiangong Chen, Xiaojing Dang, Lijun Liang, Hui Wang, Zhiyao Dong, Yang Liu, Delin Hong

**Affiliations:** State Key Laboratory of Crop Genetics and Germplasm Enhancement, Nanjing Agricultural University, Nanjing, China

**Keywords:** Rice, Grain plumpness, Association mapping, Genetic architecture, Elite allele

## Abstract

**Background:**

Poor grain plumpness (GP) is one of the main constraints to reaching the yield potential of hybrid rice.

**Results:**

In this study, the GP of 177 rice varieties was investigated in three locations across 2 years. By combining the genotype data of 261 simple sequence repeat (SSR) markers, association mapping was conducted to identify the marker-GP association loci. Among 31 marker-GP association loci detected in two or more environments and determined using general linear model (GLM) analyses, seven association loci were also detected using mixed linear model (MLM) analyses. The seven common loci detected by the two analytical methods were located on chromosomes 2, 3 (2), 7, 8 and 12 (2) and explained 7.24~22.28% of the variance. Of these 7 association loci, five markers linked to GP were newly detected: RM5340 on Chr2, RM5480 and RM148 on Chr3, RM1235 on Chr8, and RM5479 on Chr12.

**Conclusions:**

Five marker-GP association loci were newly detected using both the GLM and MLM analytical methods. Elite allele RM505-170 bp had the highest average phenotypic effect on increasing the GP, and the typical carrier variety was ‘Maozitou’. Based on the distribution of the elite alleles among the carrier varieties, the top 10 parental combinations for improving the GP in rice via cross-breeding were predicted.

**Electronic supplementary material:**

The online version of this article (10.1186/s12863-017-0559-6) contains supplementary material, which is available to authorized users.

## Background

Rice (*Oryza sativa* L.) is the main staple food for more than 50% of the world’s population [1]. As the amount of arable land area decreases, higher rice yields will be needed to meet the needs of the increasing world population [2]. The grain yield of rice per unit area of land is determined by the panicle number, grain number per panicle and grain weight. When the panicle number per unit area of land and grain number per panicle are optimized, improving the grain weight plays a key role in further increasing the yield in rice breeding programmes [3]. The grain weight is closely related to the grain size and grain plumpness (GP) [4], and the grain size is determined by the grain length, grain width and grain thickness.

Several genes have been reported to contribute to controlling grain size, including *GS3* [5, 6], *GL3* [7], *GL7* [8], *PGL1* [9] and *PGL2* [10], which regulate grain length and grain weight, and *GW2* (encoding a really interesting new gene [RING]-type E3 ubiquitin ligase) [11], *qSW5* [12], *GW5* [13], *GS5* (encoding a putative serine carboxypeptidase) [14] and *GW8* (encoding a transcription factor with a squamosa promoter binding protein-like [SBP] domain) [15], which regulate grain width and grain weight. However, poor GP remains a limiting factor for reaching the yield potential for hybrid rice, especially hybrid *japonica* rice [16, 17]. Poor GP decreases both grain weight (and thus final grain yield) and rice quality (broken grains increase after being milled). Thus, improving the GP is essential for completely realizing the yield potential of hybrid rice.

To our knowledge, 15 quantitative trait loci (QTLs) related to GP have been mapped to date, and they are distributed on chromosomes 1 (3), 2 (1), 5 (2), 6 (3), 7 (3), 8 (1), 11 (1) and 12 (1) [18–20]. However, no genes for GP have been cloned. Other studies have found that GP is closely related to sucrose synthesis and transport. Two rice sucrose synthase genes—*SUS3* on Chr7 and *SUS4* on Chr3—may be involved in carbon allocation in filling grains [21]. The cell-wall invertase gene—*OsCIN1* on Chr2—plays an important role in providing a carbon source to develop filial tissues during the early course of grain filling in the caryopsis [22]. The gene *grain incomplete filling 1* (*GIF1*) on Chr4 regulates sucrose transport and uploading during the grain-filling stage, and the overexpression of *GIF1* can increase grain filling and final grain weight [23].

Most QTLs for GP have been detected based on linkage mapping using bi-parent-derived populations, and only two alleles at a given locus have been studied [24]. Association mapping, which is a new approach, has greater power to detect more alleles or alleles with weak effects [25]. In this paper, we reported elite alleles for GP detected by association mapping using a population composed of 177 rice varieties and 261 simple sequence repeat (SSR) markers and their carrier varieties.

## Methods

### Geographical distribution of varieties used and field planting

The 177 rice varieties used in this study represent a subset of our previous reports [24, 26]. Among them, 148 were from China, and 29 were from Vietnam (Additional file 1: Table S1). The varieties were distributed from 17.00°N to 41.81°N. The 177 varieties were grown from May to November 2013 and 2014 at three locations: Jiangpu Experimental Farm (JEF; 118.62°E, 32.07°N), Nanjing Agricultural University, Jiangsu province, and Xinyang Farm (XF; 114.12°E, 32.10°N) and Yuanyang Farm (YF; 113.96°E, 35.05°N), Henan Academy of Agricultural Sciences, Henan Province, China. JEF and XF are located at almost the same latitude, but their longitudes differ by 4.5°. XF and YF are located at almost the same longitude, but their latitudes differ by 3°. Seedlings aged approximately 30 days were transplanted to the paddy field by hand each year at each location. Each plot consisted of five rows with eight plants per row, and the spacing was 17 cm × 20 cm. The field trial was arranged using a completely randomized block design with two replications at each location.

### Phenotyping

The main stem panicles of the 10 plants in the middle three rows of each plot were harvested, threshed and dried under natural sunshine to 13% moisture. All dried spikelets were placed on a translucent lamp box, and the empty grains (unfertilized spikelets) were selected by hand. Then, the full grains were separated from the remaining mixed filled grains (full plus shrunken) using a salt solution with a specific gravity of 1.1. The full grains and shrunken grains were then dried at 105 °C for 24 h to constant weight. The measurements of the full grains and mixed filled grains for each plot were replicated three times. The GP was calculated using the following formula:

$$ GP\left(\%\right)=\frac{A_c}{B_c}\times 100 $$

where A_c_ represents the average thousand-grain weight of the mixed filled grains of variety c, and B_c_ represents the average thousand-grain weight of the full grains of variety c [27].

### Genotyping

The SSR molecular marker genotype data published in [26] were used in this study, except for RM433 on chromosome 8, which showed no polymorphism among the 177 accessions. The base pair start positions on the chromosomes for each SSR marker are presented (Additional file 2: Table S2) for calculating the physical distance between markers on the chromosomes.

### Data analysis

The phenotypic data were statistically analysed using Microsoft Excel 2010. The broad-sense heritability was computed using the formula [28]

$$ {H^2}_B={\sigma^2}_g/\left({\sigma^2}_g+{\sigma^2}_e/n\right) $$

where σ^2^_g_ is the genetic variance, σ^2^_e_ is the error variance, and n is the number of replications.

Two methods were used to detect the population genetic architecture of the 177 accessions. The first was the Bayesian cluster analysis approach, which was implemented using STRUCTURE version 2.2 [29]. The second was the neighbour-joining method, which was carried out using MEGA version 5.0 based on Nei’s genetic distance [30]. The computations followed the same approach as those described in [31]. The coefficient of genetic differentiation (*F*_ST_) [32] was calculated to measure the fixation of different alleles in different subpopulations using Arlequin version 3.0 [33]. The number of alleles per locus, gene diversity and polymorphism information content (PIC) were determined using PowerMarker version 3.25. The *r* ^2^ value [34] calculated via TASSEL version 2.1 [35] was used as the preferred measure of linkage disequilibrium (LD).

Two models, the general linear model (GLM) and the mixed linear model (MLM), were used to analyse the associations between GP and SSR markers with TASSEL version 2.1. In the GLM, only the Q matrix was used as a covariate, while in the MLM, both the Q matrix and kinship matrix were used as covariates [36]. The kinship matrix was calculated via SPAGeDi to estimate the genetic relatedness among individuals [37]. A false discovery rate (FDR) of 0.01 was used as a threshold for significant associations [38]. Based on the identified association locus, the ‘null allele’ (non-amplified allele) was used to determine the phenotypic effects of other alleles [39]. Alleles with frequencies of less than 5% in the population were regarded as rare alleles and treated as missing data. The following formula was used to calculate the positive (negative) average allele effect (AAE) of each locus:

$$ AAE=\sum {a}_i/{n}_i $$

where ∑a_i_ is the positive (negative) allelic phenotypic effects of locus i, and n_i_ is the number of positive (negative) alleles within locus i.

## Results

### Phenotypic variation and genetic diversity in the population studied

Among the six environments, the mean GP values were higher than 90%, and the coefficient of variation ranged from 3.35% to 4.22%. The broad-sense heritability for the GP was greater than 90% in each environment (Table 1). No significant differences were detected over 2 years at any location, indicating that the GP is influenced mainly by genetic factors. A two-way analysis of variance (ANOVA) showed that the differences in GP among the 177 varieties were significant at the α = 0.01 probability level, indicating that a large amount of genetic variation existed in the entire population.

**Table 1:** Descriptive statistics for GP (%) for two years at three locations

Location	Year	Mean ± SD^a^	Maximum	Minimum	CV (%)^b^	*H* ^*2*^_B_ (%)^c^
YF^d^	2013	94.33 ± 3.68	99.51	84.89	3.91	97.09
	2014	93.47 ± 3.77	99.52	79.43	4.04	98.61
XF^e^	2013	92.20 ± 3.89	99.33	77.89	4.22	96.75
	2014	90.31 ± 3.72	98.37	78.23	4.12	96.45
JEF^f^	2013	93.02 ± 3.20	99.55	82.66	3.44	95.22
	2014	93.12 ± 3.12	99.17	83.25	3.35	96.95

^a^ *SD* Standard deviation, ^b^ *CV* Coefficient of variation, ^c^ *H* ^*2*^_*B*_ Broad-sense heritability, ^d^ *YF* Yuanyang Farm, ^e^ *XF* Xinyang Farm, ^f^ *JEF* Jiangpu Experimental Farm

Highly significant correlations (α = 0.01) were observed for the GP trait between the 2 years at each location. The coefficients of correlation between the 2 years were 0.814 at YF, 0.975 at XF and 0.974 at JEF. The coefficients of correlation between the pairs of locations were 0.432 (r^JEF-XF^, JEF and XF), 0.312 (r^YF-XF^, YF and XF) and 0.367 (r^YF-JEF^, YF and JEF). Thus, the variation tendency of the GP was consistent across years and locations.

Of the 1948 alleles amplified by 261 SSR marker loci in the 177 varieties, 35.14% were rare alleles with frequencies less than 5%. The average number of alleles per SSR locus was 7.46 and ranged from 2 to 20. The average gene diversity was 0.6734 and ranged from 0.0223 (RM140 on Chr1) to 0.9152 (RM7545 on Chr10). The average PIC value was 0.6395 and ranged from 0.0221 (RM140 on Chr1) to 0.9091 (RM7545 on Chr10) (Additional file 2: Table S2).

### Genetic architecture of the original population

**Fig. 1:**
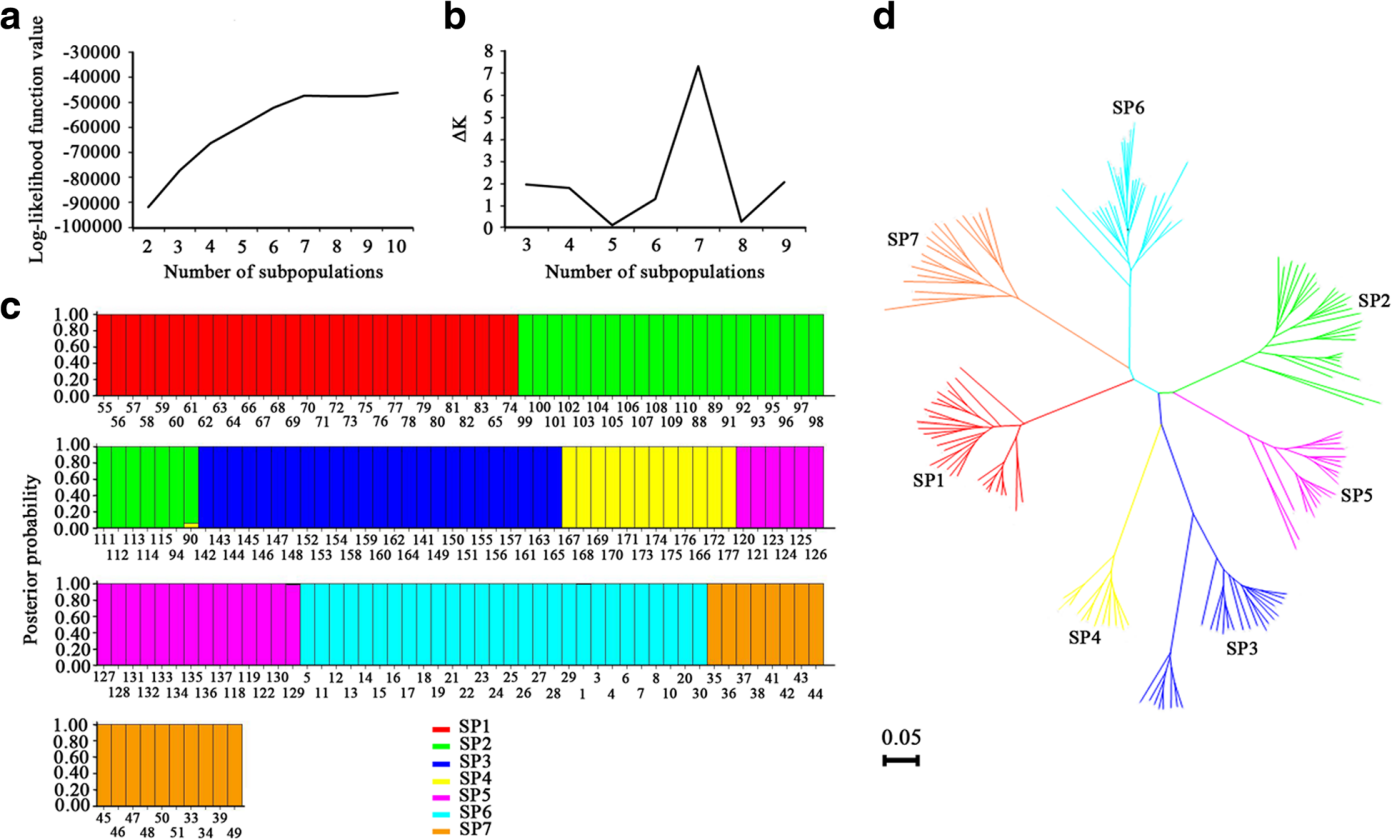
Population genetic architecture analysis of 160 varieties. Effects of changes in the log-likelihood function value (**a**) and the ΔK value (**b**) on the number of subpopulations, posterior probabilities of 160 varieties belonging to seven subpopulations (**c**) and the neighbour-joining tree for 160 varieties based on Nei’s genetic distance (**d**). Each variety is represented by a vertical bar. The coloured subsections within each vertical bar indicate the membership coefficients (Q) of that variety in different subpopulations. The identified subpopulations are SP1 (*red*), SP2 (*green*), SP3 (*navy blue*), SP4 (*yellow*), SP5 (*purple*), SP6 (*light blue*), and SP7 (*brown*)

Although the 177 accessions represent a subset of the 540 accessions used in our previous reports [26], the present population was still divided into seven subpopulations using ΔK as the diagnostic criterion (Additional file 3: Figure S1a and 1b). This may be caused by a broad geographical distribution (17.00°N to 41.81°N), although the number of points was reduced. Based on the criterion of Q > 0.900, 17 varieties were assigned to the admixed group and were not analysed further. The other 160 varieties were reanalysed using the STRUCTURE software package, and they were clearly differentiated into seven subpopulations with Q > 0.900 for each variety (Fig. 1c; Additional file 1: Table S1). The neighbour-joining tree constructed based on Nei’s genetic distance [30] supported the finding that the original population was composed of seven subpopulations (i.e., SP1 to SP7; Fig. 1d). The numbers of varieties included in SP1-SP7 were 29, 28, 25, 12, 20, 28 and 18, respectively. The varieties in SP3 and SP4 are mainly from Vietnam, whereas the varieties in the other five subpopulations are from China (Additional file 1: Table S1).

### Pairwise *F*_ST_ and Nei’s genetic distance among subpopulations

The average *F*_ST_ value of the seven subpopulations (160 varieties) was 0.6587. The *F*_ST_ value between SP3 and SP4 was the lowest (0.5978), while that between SP1 and SP4 was the highest (0.7451). Nei’s genetic distance between SP3 and SP1 was the longest (0.7624), whereas that between SP5 and SP2 was the shortest (0.5032) (Table 2). In addition, when the *F*_ST_ value between SP3 and SP4 was lowest, Nei’s genetic distance between SP3 and SP4 was shorter, whereas when the *F*_ST_ between SP1 and SP4 was highest, Nei’s genetic distance between SP1 and SP4 was longer (Table 2). These findings reveal that the pairwise *F*_ST_ can reflect the genetic distance between subpopulations.

**Table 2:** Pairwise *F*_ST_ and Nei’s genetic distance among the seven subpopulations

Subpopulation	SP1	SP2	SP3	SP4	SP5	SP6	SP7
SP1	–	0.6347	0.6673	0.7451	0.6875	0.6437	0.6671
SP2	0.5548	–	0.6264	0.6933	0.6026	0.6304	0.6446
SP3	0.7624	0.6821	–	0.5978	0.6388	0.6438	0.6235
SP4	0.7465	0.6612	0.5576	–	0.7302	0.7072	0.7056
SP5	0.6207	0.5032	0.6792	0.6607	–	0.6591	0.6743
SP6	0.5844	0.6107	0.7482	0.7151	0.6235	–	0.6109
SP7	0.6581	0.6664	0.7542	0.7346	0.6884	0.5558	–

Nei’s genetic distance is below the diagonal, and *F*_ST_ is above the diagonal. All pairwise *F*_ST_ values are significant (*P* < 0.01)

### Significant LD of SSR markers in the seven subpopulations

Table 3 shows the levels of LD estimated for the entire population and the seven subpopulations. For the entire population, the percentage of significant (*P* < 0.01) locus pairs in LD among the total number of locus pairs was 95.21%. The percentage for pairs of markers from the same chromosome was 95.97%, higher than the value determined for markers from different chromosomes (95.14%). Of the seven subpopulations, SP4 had the lowest percentage (2.35%), and SP3 had the highest percentage (44.94%). The 75th percentiles for the background LD values of the seven subpopulations were 0.0785, 0.0807, 0.1065, 0.0988, 0.0994, 0.0662 and 0.0982, respectively (Additional file 4: Figure S2). A regression analysis of the *r* ^2^ value and the genetic distance for the linked SSR marker pairs showed that the LD decay distances for SP1-SP7 were 4.48 Mb, 5.48 Mb, 4.26 Mb, 3.53 Mb, 5.53 Mb, 5.58 Mb and 8.67 Mb, respectively, under the background LD (Additional file 5: Figure S3). This result further validated the significant LD of the SSR markers in the seven subpopulations and demonstrated that the LD decay velocity varied among these subpopulations.

**Table 3:** Percentages of significant (*P* < 0.01) locus pairs in LD

Sub-population	Markers on the same chromosome		Markers on different chromosomes		Total	
	No. of locus pairs in LD^a^	Percentage of locus pairs (%)	No. of locus pairs in LD^a^	Percentage of locus pairs (%)	No. of locus pairs in LD^a^	Percentage of locus pairs (%)
SP1	77 (958)	8.04	857 (10532)	8.14	934 (11490)	8.13
SP2	107 (1449)	7.38	1226 (16454)	7.45	1333 (17903)	7.45
SP3	495 (1048)	47.23	5265 (11768)	44.74	5760 (12816)	44.94
SP4	11 (353)	3.12	93 (4065)	2.29	104 (4418)	2.35
SP5	49 (1090)	4.50	566 (11782)	4.80	615 (12872)	4.78
SP6	60 (1047)	5.73	583 (11763)	4.96	643 (12810)	5.02
SP7	46 (1048)	4.39	538 (12074)	4.46	584 (13122)	4.45
All	1903 (1983)	95.97	20811 (21874)	95.14	22,714 (23857)	95.21

^a^The values in parentheses are the total numbers of locus pairs

### Significant SSR marker-GP association loci detected in the population studied

In total, 31 association loci between the SSR marker and GP with *P*-values less than 0.01 were detected by both the GLM and MLM analyses in two or more environments (Additional file 6: Table S3). The GLM analysis revealed 31 marker loci associated with GP (*P* < 0.01), and the identified markers were located on all of the chromosomes except for chromosome 11. The rate of phenotypic variation explained (PVE) ranged from 7.24% to 22.28%. RM5479 on Chr12 exhibited the highest PVE values: 22.28% in 2013 and 20.64% in 2014 (Additional file 6: Table S3). The MLM analysis revealed seven marker loci associated with GP (*P* < 0.01) located on chromosomes 2, 3 (2), 7, 8 and 12 (2). The PVE ranged from 9.01% to 21.06%. RM5479 on Chr12 also exhibited the highest PVE values: 21.06% in 2013 and 20.39% in 2014 (Additional file 6: Table S3). Seven marker loci—RM5340 on Chr2, RM5480 on Chr3, RM148 on Chr3, RM505 on Chr7, RM1235 on Chr8, RM511 on Chr12 and RM5479 on Chr12—were detected by both the GLM and MLM analyses. Of these seven association loci, RM505 on Chr7 had the highest positive AAE value (1.68%).

### Elite alleles for GP

The seven common marker-GP association loci from both the GLM and MLM analyses were considered to be robust loci associated with GP (Additional file 6: Table S3). Based on these seven markers, 15 elite alleles were mined in two or more environments (Table 4). RM505-170 bp had an average phenotypic effect of increasing the GP by 2.62%, and the typical carrier variety was ‘Maozitou’. RM5479-215 bp exhibited the second highest average phenotypic effect of 2.48%, and the carrier variety was ‘Zaoguangtou’. Some varieties carried several elite alleles, such as ‘Yuedao 5’, indicating that they are excellent donor varieties for improving GP.

**Table 4:** Elite alleles with positive phenotypic effects for GP and typical carrier varieties

Marker	PVE^a^ (%)	Elite allele (bp)	Phenotypic effect value (%)							Typical carrier variety
			YF^b^		XF^c^		JEF^d^		Mean	
			2013	2014	2013	2014	2013	2014		
RM5340	16.7	95	0.29	0.65					0.47	Yuedao 5
		155	1.94	1.77					1.86	Yazihuang
		205	2.36	2.51					2.44	Yuedao 22
RM5480	8.42	165			1.8		1.72	1.86	1.79	Yuedao 5
		200			0.87		1.75	1.34	1.32	Ligengqing
RM148	9.93	125			1.34	1.29			1.32	Huangsanshi
RM505	17.13	160			2.15	1.76			1.96	Qiutiandaxiedao
		170			2.74	2.49			2.62	Maozitou
		180			0.47	0.47			0.47	Chiguhong
RM1235	8.76	120		0.52		1.63	1.63		1.26	Yuedao 5
RM511	13.15	130					2.48	2.3	2.39	Qiaobinghuang
		135					0.32	0.25	0.28	Yuedao 5
RM5479	21.46	210					1.77	1.88	1.83	Ligengqing
		215					2.72	2.23	2.48	Zaoguangtou
		225					0.58	0.83	0.7	Yuedao 5

^a^ *PVE* Percentage of phenotypic variation explained, ^b^ *YF* Yuanyang Farm, ^c^ *XF* Xinyang Farm, ^d^ *JEF* Jiangpu Experimental Farm

### Top parental combinations predicted for GP improvement

**Fig. 2:**
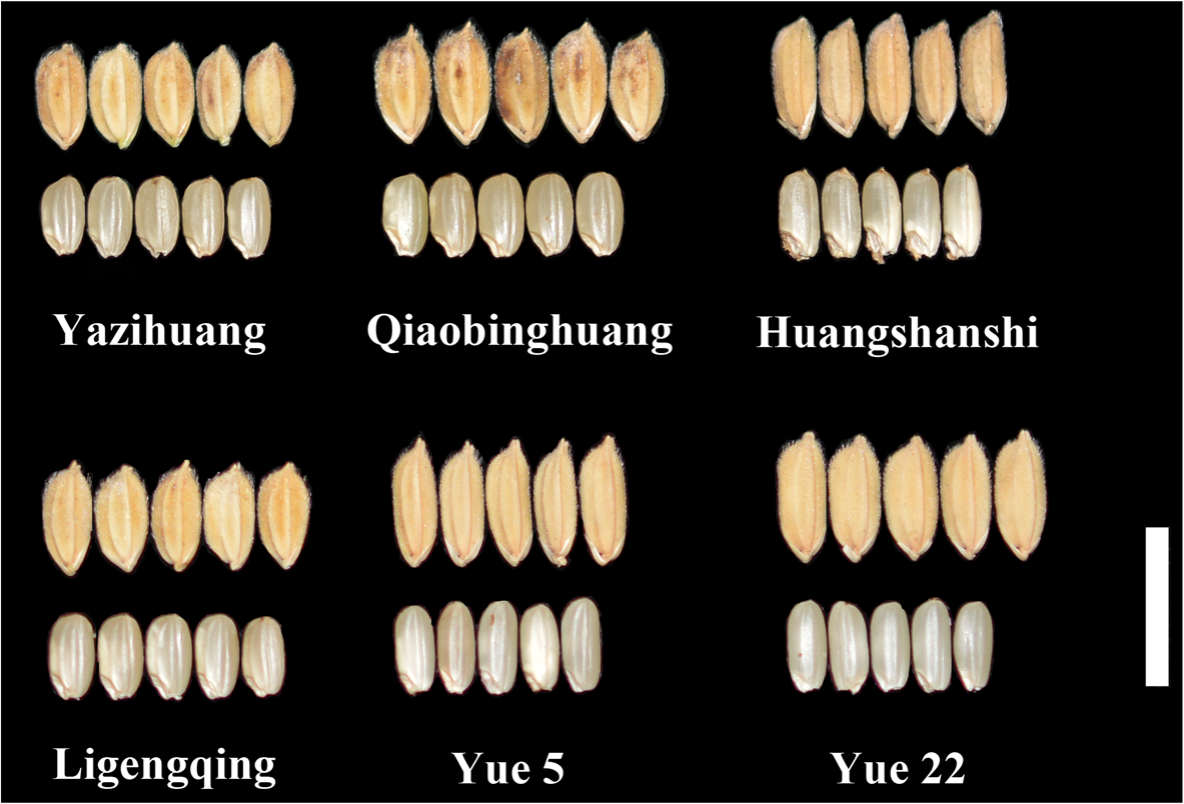
Unhulled rice grains and brown rice grains of the investigated varieties in the predicted combinations for GP improvement (bar = 1 cm)

Based on the data in Additional file 6: Table S3, the alleles at seven significant marker-GP association loci in typical carrier varieties were analysed (Additional file 3: Table S3). The top 10 parental combinations were predicted (Table 5) for improving the GP in rice via cross-breeding based on the data presented in Additional file 3: Table S3. For instance, ‘Yuedao 5’ had six elite alleles, and ‘Ligengqing’ had six elite alleles. Seven elite alleles could be pyramided into one plant using the combination ‘Yuedao 5 × Ligengqing’, and as a result, the GP should theoretically be improved by 13.07% (Additional file 7: Table S4; Table 5). Figure 2 shows the unhulled rice grains and brown rice grains of the varieties corresponding to the predicted combinations to improve GP.

**Table 5:** Parental combinations predicted for GP improvement

Parental combination predicted	Number of elite alleles predicted	GP improvement predicted (%)
Yuedao 5 × Ligengqing	7	13.07
Yuedao 22 × Ligengqing	7	13.07
Yazihuang × Ligengqing	7	13.07
Yazihuang × Huangsanshi	7	13.07
Yuedao 22 × Yuedao 5	7	12.52
Yazihuang × Yuedao 22	7	12.52
Yuedao 5 × Zaoguangtou	7	12.33
Yuedao 5 × Huangsanshi	7	11.68
Yuedao 5 × Qiaobinghuang	7	10.55
Yuedao 5 × Yazihuang	6	8.37

## Discussion

GP in rice affects not only yield but also milling quality (recovery of head rice), especially in hybrid rice where heavy panicles often increase the yield [17, 40]. Mining elite alleles for GP is beneficial for improving this trait. In the present study, we used 177 accessions, representing a subset of the population (540 accessions) reported in [26], and 261 SSR markers to implement the discovery. To avoid spurious associations in association mapping [41], we first evaluated the present population genetic architecture using two different analysis methods (STRUCTURE and Nei’s genetic distance) and detected 7 subpopulations (Fig. 1), which was the same as those detected in [26]. We inferred that the population genetic structure was mainly affected by geographical location (ecotypes) and nearly unaffected by accession reducing.

**Fig. 3:**
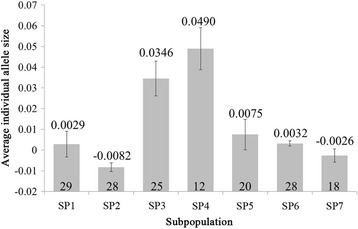
Average standardized individual allele sizes of the seven subpopulations. The mean, standard deviation, and number of varieties per subpopulation are identified

We also found that the LD decay distances for SP1-SP7 were 4.48 Mb, 5.48 Mb, 4.26 Mb, 3.53 Mb, 5.53 Mb, 5.58 Mb and 8.67 Mb, respectively. The fast decays in SP3 and SP4 could have resulted from rapid artificial hybridization in Vietnam, which should accelerate the recombination of the chromosomes and, thereby, weaken the LD. We calculated the average standardized individual allele sizes of the seven subpopulations according to the methods described by [42] and observed that the average allele sizes in SP3 and SP4 were significantly higher than those in the other subpopulations (Fig. 3). This fact further confirmed that directional evolution for the allele size has occurred in rice [24, 31]. We also observed that the SSR alleles tended to decrease in size from the low-latitude subpopulations (SP3 and SP4, 17–23゜N) to the high-latitude subpopulations (the other five subpopulations, 30–39゜N); this behaviour may be explained by the emergence of mutations or changes in the mutation rate causing a change in the allele size in rice [43, 44]. No significant differences were found between SP3 and SP4, possibly because of the short geographical distance between the two subpopulations. The same phenomenon was detected among the remaining five subpopulations (Table 6). Moreover, the high proportion of the rare alleles (35.14%) might be related to the geography of rice migration. New alleles appeared and certain original alleles disappeared with the changes in the cultivation environment, resulting in the emergence of varieties with rare alleles.

**Table 6:** Differences in the average standardized individual allele sizes among the seven subpopulations

Subpopulation	SP1	SP2	SP3	SP4	SP5	SP6	SP7
SP1							
SP2	−1.51						
SP3	3.98**	6.21**					
SP4	4.41**	6.44**	2.02				
SP5	0.37	1.32	−2.89**	−3.67**			
SP6	0.05	1.25	−4.09**	−4.24**	−0.32		
SP7	−0.48	0.45	−4.30**	−4.39**	−0.59	−0.53	

**indicates that the difference between two subpopulations is significant at *P* < 0.01

Furthermore, the number of detected marker-GP association loci decreased when the same population was cultivated towards the west and north. As shown in Additional file 6: Table S3, in JEF (32.07°N, 118.62°E), 24 and 21 marker-GP association loci were detected in 2013 and 2014, respectively, by the GLM analyses, and four (RM5480, RM1235, RM511 and RM5479) and three (RM5480, RM511 and RM5479) association markers were detected in 2013 and 2014, respectively, by the MLM analyses. In XF (32.10°N, 114.12°E), ten and six marker-GP association loci were detected in 2013 and 2014, respectively, by the GLM analyses, and two (RM148 and RM505) and three (RM148, RM505 and RM1235) association markers were detected in 2013 and 2014, respectively, by the MLM analyses. In YF (35.05°N, 113.96°E), four and seven SSR marker-GP association loci were detected in 2013 and 2014, respectively, by the GLM analyses, and only one association marker (RM5340) was detected by the MLM analyses in the both years. Furthermore, among the seven association loci detected by the two analytical methods (GLM and MLM), no identical association marker loci were found among the three locations except for RM1235 on Chr8. Thus, there are many gene loci underlying GP, and different genes exhibit different characteristics in different environments.

Based on previous studies, *GIF1*, as an important gene cloned associated with rice grain filling, encodes a cell-wall invertase required for carbon partitioning during early grain filling. *GIF1* is a potential domestication gene; thus, a domestication-selected gene can be used for further crop improvement. In our study, we found no markers associated with GP near the region of *GIF1*. By comparing with other studies, we found that five of the seven SSR markers detected in this study were novel; the other two SSR markers were located near the chromosome regions harbouring grain filling and related QTLs or genes reported in previous studies (Additional file 8: Table S5).Among the seven SSR markers, RM505 on Chr7 was in the region of *qGR-7-8* for grain-filling rate [20], and RM511 on Chr12 was the same as the SSR markers for grain-filling rate detected by [45], implying that GP was affected by the grain milk filling rate.

Although the PVE of the seven association loci ranged from 7.24% to 22.28%, the positive AAE values were weak (1.26% on RM1235 to 1.68% on RM505). This result might be explained by the coefficient of variation in GP in the population was not large enough (CV < 5%, Table 1) because the experimental materials were cultivars in different areas. However, because GP is a function of grain weight, a small improvement will contribute considerably to the grain yield of rice.

The elite alleles mined in this study may be used to improve the GP of hybrid rice. Among the top 10 parental combinations predicted, the combinations ‘Yuedao 5 × Ligengqing’, ‘Yuedao 22 × Ligengqing’, ‘Yazihuang × Ligengqing’ and ‘Yazihuang × Huangsanshi’ could theoretically improve the GP by 13.07% (Table 5). Six of the ten combinations have ‘Yuedao 5’ as a parent, indicating that ‘Yuedao 5’ may be an excellent parent for GP improvement.

## Conclusions

Seven marker loci were detected for GP, of which five were novel loci. Ten parental combinations were predicted for improving the GP in rice via cross-breeding.

## Additional files

Additional file 1: Table S1. Origins and Q values of the varieties used in the present study. All varieties used in this study were pure line varieties. Bold varieties have Q < 0.900 and could not be assigned to any subpopulation. (XLS 82 kb)

Additional file 2: Table S2. Summary statistics for the 261 SSR markers used in the present study. (DOCX 56 kb)

Additional file 3: Figure S1. Population genetic architecture analysis of 177 varieties. Effects of changes in log-likelihood function value (a) and the ΔK value (b) on the number of subpopulations and the posterior probabilities of 177 varieties belonging to seven subpopulations (c). Each variety is represented by a vertical bar. The coloured subsections within each vertical bar indicate the membership coefficients (Q) of each variety in different subpopulations. The identified subpopulations are sp1 (*red*), sp2 (*green*), sp3 (*navy blue*), sp4 (*yellow*), sp5 (*purple*), sp6 (*light blue*), and sp7 (*brown*). (TIFF 291 kb)

Additional file 4: Figure S2. Distribution of the linkage disequilibrium *r* ^2^ values between the unlinked SSRs for the seven subpopulations. The 75th percentiles of the *r* ^2^ values for the seven subpopulations are indicated. (TIFF 287 kb)

Additional file 5: Figure S3. Relationship between the *r* ^2^ value and genetic distance for the linked SSR marker pairs for the seven subpopulations. The horizontal line indicates the 75th percentile determined for the distribution of the unlinked SSRs. (TIFF 766 kb)

Additional file 6: Table S3. Marker-trait association loci with *P* < 0.01 determined by the GLM and MLM analyses, their FDRs, proportions of phenotypic variance explained, AAE values, and marker positions on the chromosome derived for 261 markers and 160 rice varieties. (DOCX 27 kb)

Additional file 7: Table S4. Alleles at seven maker-trait loci in typical carrier varieties. ‘√’ indicates that the variety has the positive allele, and ‘∆’ indicates that the variety has the negative allele. (DOCX 22 kb)

Additional file 8: Table S5. Comparison of SSR markers identified in this study and genes or QTLs reported in previous studies. The physical positions of the third and fifth columns were determined based on data from Gramene (http://www.gramene.org/markers) and NCBI (http://blast.ncbinlm.nih.gov/Blast.cgi). (DOCX 19 kb)

Additional file 9: Table S6. Grain plumpness of 177 rice accessions at three locations in 2013 and 2014. (XLSX 148 kb)

Additional file 10: Table S7. Genotypes of 177 rice accessions with 261 SSR markers on 12 chromosomes. (XLSX 244 kb)

## Abbreviations

AAE: Average allele effect
ANOVA: Analysis of variance
FDR: False discovery rate
F_*ST*_: Coefficient of genetic differentiation
GLM: General linear model
GP: Grain plumpness
H^2^_B_: Broad-sense heritability
LD: Linkage disequilibrium
MLM: Mixed linear model
PIC: Polymorphism information content
PVE: Phenotypic variation explained
SSR: Simple sequence repeat
