# Supplementary material for: Identification of putative markers linked to grain plumpness in rice (Oryza sativa L.) via association mapping
Source: BMC Genet. 2017 Oct 12;18:89. doi: 10.1186/s12863-017-0559-6 (PMC5639755; doi:10.1186/s12863-017-0559-6)
Supplement: Supplementary file 2 — Summary statistics for the 261 SSR markers used in the present study. (DOCX 56 kb) [file 12863_2017_559_MOESM2_ESM.docx]

**Table S2. Summary statistics for the 261 SSR markers used in the present study**

| Locus | Chr.No. | Start position | Allele number | Gene Diversity | PIC^a^ | Locus | Chr.No. | Start position | Allele number | Gene Diversity | PIC^a^ |
| --- | --- | --- | --- | --- | --- | --- | --- | --- | --- | --- | --- |
|  |  | (bp) |  |  |  |  |  | (bp) |  |  |  |
| RM84 | 1 | 3945672 | 6 | 0.3111 | 0.2996 | RM136 | 6 | 8763322 | 12 | 0.7964 | 0.7781 |
| RM1-003 | 1 | 4052832 | 9 | 0.8203 | 0.8012 | RM3330 | 6 | 10907997 | 10 | 0.7571 | 0.7286 |
| RM1 | 1 | 4636793 | 6 | 0.761 | 0.7246 | RM3187 | 6 | 20579001 | 6 | 0.7272 | 0.677 |
| RM283 | 1 | 4885944 | 7 | 0.7714 | 0.7369 | RM7579 | 6 | 23119353 | 5 | 0.6566 | 0.5971 |
| RM3453 | 1 | 4887046 | 10 | 0.8232 | 0.8015 | RM454 | 6 | 23381619 | 4 | 0.4613 | 0.4284 |
| RM490 | 1 | 6676165 | 4 | 0.2271 | 0.2178 | RM345 | 6 | 23381619 | 5 | 0.4151 | 0.366 |
| RM259 | 1 | 7445746 | 13 | 0.842 | 0.8238 | RM162 | 6 | 23652879 | 12 | 0.8677 | 0.8535 |
| RM583 | 1 | 8328958 | 6 | 0.7037 | 0.6513 | RM8239 | 6 | 24555002 | 4 | 0.7236 | 0.6731 |
| RM8095 | 1 | 11238348 | 7 | 0.7785 | 0.7431 | RM7309 | 6 | 25914707 | 9 | 0.7457 | 0.7203 |
| RM140 | 1 | 12284725 | 2 | 0.0223 | 0.0221 | RM528 | 6 | 26172237 | 8 | 0.5879 | 0.5302 |
| RM562 | 1 | 14610402 | 14 | 0.8925 | 0.8836 | RM3138 | 6 | 28086462 | 11 | 0.7939 | 0.7699 |
| RM129 | 1 | 19005894 | 2 | 0.0863 | 0.0826 | RM6811 | 6 | 28846137 | 13 | 0.889 | 0.8787 |
| RM9 | 1 | 23322208 | 10 | 0.8294 | 0.8093 | RM5753 | 6 | 30452023 | 14 | 0.8568 | 0.8419 |
| RM5 | 1 | 23968577 | 9 | 0.8489 | 0.8308 | RM295 | 7 | 414668 | 10 | 0.6771 | 0.6502 |
| RM246 | 1 | 27329504 | 9 | 0.7636 | 0.7314 | RM82 | 7 | 3128276 | 4 | 0.2783 | 0.2621 |
| RM1231 | 1 | 29472375 | 12 | 0.8622 | 0.8474 | RM8263 | 7 | 4688095 | 8 | 0.6934 | 0.6634 |
| RM128 | 1 | 30732043 | 14 | 0.7809 | 0.7633 | RM125 | 7 | 5480476 | 3 | 0.5888 | 0.5006 |
| RM297 | 1 | 32093949 | 5 | 0.5938 | 0.5166 | RM180 | 7 | 5768455 | 8 | 0.6846 | 0.6328 |
| RM212 | 1 | 33047896 | 3 | 0.2066 | 0.1961 | RM542 | 7 | 12659507 | 7 | 0.7821 | 0.7468 |
| RM486 | 1 | 34949898 | 8 | 0.7604 | 0.7211 | RM2530 | 7 | 15570383 | 10 | 0.8492 | 0.8323 |
| RM265 | 1 | 35197706 | 5 | 0.7374 | 0.6924 | RM418 | 7 | 18079728 | 8 | 0.568 | 0.5382 |
| RM5389 | 1 | 35726691 | 10 | 0.8402 | 0.8206 | RM11 | 7 | 19258015 | 6 | 0.5905 | 0.5091 |
| RM3482 | 1 | 39713330 | 12 | 0.7885 | 0.7721 | RM5380 | 7 | 19308732 | 8 | 0.7469 | 0.704 |
| RM14 | 1 | 41363934 | 10 | 0.8401 | 0.8201 | RM6011 | 7 | 20733917 | 10 | 0.8525 | 0.8349 |
| RM6831 | 1 | 43171288 | 4 | 0.5913 | 0.5258 | RM346 | 7 | 21045102 | 9 | 0.7644 | 0.7335 |
| RM5340 | 2 | 7482346 | 13 | 0.8436 | 0.8274 | RM336 | 7 | 21818658 | 9 | 0.7096 | 0.6789 |
| RM7288 | 2 | 9032703 | 14 | 0.829 | 0.8089 | RM505 | 7 | 24527931 | 6 | 0.7833 | 0.7506 |
| RM5356 | 2 | 9431672 | 7 | 0.738 | 0.6937 | RM3589 | 7 | 25054610 | 11 | 0.7905 | 0.764 |
| RM1358 | 2 | 10184719 | 6 | 0.769 | 0.7298 | RM234 | 7 | 25420132 | 10 | 0.8346 | 0.8154 |
| RM1313 | 2 | 11262096 | 9 | 0.7674 | 0.7409 | RM473B | 7 | 25458227 | 8 | 0.8223 | 0.7997 |
| RM324 | 2 | 11388913 | 4 | 0.3638 | 0.3317 | RM134 | 7 | 26584000 | 6 | 0.7751 | 0.7434 |
| RM301 | 2 | 12216596 | 7 | 0.7924 | 0.7657 | RM1306 | 7 | 28894398 | 8 | 0.7391 | 0.7024 |
| RM300 | 2 | 13190528 | 8 | 0.8214 | 0.7963 | RM506 | 8 | 126285 | 10 | 0.7683 | 0.7356 |
| RM6361 | 2 | 17136965 | 5 | 0.71 | 0.6571 | RM1019 | 8 | 196019 | 12 | 0.7853 | 0.7648 |
| RM327 | 2 | 20057306 | 5 | 0.781 | 0.746 | RM152 | 8 | 677702 | 9 | 0.81 | 0.7841 |
| RM262 | 2 | 20800840 | 6 | 0.7357 | 0.6947 | RM1235 | 8 | 1203431 | 5 | 0.5995 | 0.5278 |
| RM5427 | 2 | 21544386 | 6 | 0.5224 | 0.4728 | RM6863 | 8 | 2005990 | 10 | 0.7875 | 0.7614 |
| RM3688 | 2 | 22420221 | 8 | 0.8293 | 0.8068 | RM25 | 8 | 4378457 | 9 | 0.77 | 0.7338 |
| RM5804 | 2 | 24614505 | 6 | 0.7097 | 0.6604 | RM4085 | 8 | 4443963 | 12 | 0.8777 | 0.8651 |
| RM106 | 2 | 25147239 | 12 | 0.8427 | 0.8255 | RM544 | 8 | 5102982 | 8 | 0.8643 | 0.8489 |
| RM263 | 2 | 25889828 | 12 | 0.8778 | 0.8653 | RM80 | 8 | 5768455 | 11 | 0.6688 | 0.6446 |
| RM573 | 2 | 27965302 | 14 | 0.8407 | 0.8226 | RM72 | 8 | 6763705 | 12 | 0.7993 | 0.7807 |
| RM525 | 2 | 28292005 | 12 | 0.7362 | 0.7096 | RM8243 | 8 | 8930116 | 7 | 0.8061 | 0.7791 |
| RM450 | 2 | 28652656 | 6 | 0.7668 | 0.7375 | RM331 | 8 | 12288130 | 8 | 0.7935 | 0.7624 |
| RM7598 | 2 | 29846409 | 5 | 0.5076 | 0.4747 | RM6215 | 8 | 19058582 | 9 | 0.798 | 0.7757 |
| RM112 | 2 | 32019760 | 5 | 0.7477 | 0.707 | RM7556 | 8 | 22202567 | 5 | 0.7697 | 0.7328 |
| RM6114 | 2 | 32597667 | 8 | 0.8438 | 0.824 | RM6976 | 8 | 23551198 | 12 | 0.82 | 0.7992 |
| RM213 | 2 | 34676726 | 8 | 0.829 | 0.8074 | RM502 | 8 | 26487741 | 8 | 0.7921 | 0.7625 |
| RM208 | 2 | 35160202 | 4 | 0.5983 | 0.522 | RM3754 | 8 | 26965784 | 7 | 0.7469 | 0.7183 |
| RM498 | 2 | 35424368 | 11 | 0.7983 | 0.7699 | RM6948 | 8 | 27305525 | 4 | 0.7114 | 0.6629 |
| RM266 | 2 | 35431738 | 7 | 0.7082 | 0.662 | RM281 | 8 | 27891048 | 9 | 0.6909 | 0.6384 |
| RM3850 | 2 | 35450187 | 13 | 0.8604 | 0.8451 | RM264 | 8 | 27926632 | 9 | 0.8582 | 0.8414 |
| RM48 | 2 | 35535192 | 3 | 0.2426 | 0.2273 | RM8206 | 9 | 5866359 | 7 | 0.748 | 0.7105 |
| RM535 | 2 | 35802733 | 9 | 0.802 | 0.7772 | RM1328 | 9 | 9152293 | 12 | 0.8678 | 0.8549 |
| RM132 | 3 | 995071 | 5 | 0.4905 | 0.4509 | RM3912 | 9 | 10774451 | 8 | 0.8328 | 0.81 |
| RM1332 | 3 | 2435994 | 4 | 0.6873 | 0.622 | RM524 | 9 | 12871621 | 9 | 0.8195 | 0.7964 |
| RM5849 | 3 | 4237901 | 14 | 0.7837 | 0.7543 | RM566 | 9 | 14651176 | 6 | 0.7058 | 0.6558 |
| RM489 | 3 | 4316616 | 8 | 0.8228 | 0.8003 | RM434 | 9 | 15609041 | 5 | 0.7791 | 0.7435 |
| RM545 | 3 | 4916484 | 2 | 0.4518 | 0.3498 | RM3600 | 9 | 17054142 | 6 | 0.6932 | 0.6439 |
| RM5480 | 3 | 5306122 | 5 | 0.6124 | 0.567 | RM24481 | 9 | 17210236 | 13 | 0.8561 | 0.8403 |
| RM3467 | 3 | 5972018 | 11 | 0.8158 | 0.7946 | RM410 | 9 | 17589271 | 6 | 0.711 | 0.6611 |
| RM3766 | 3 | 6902135 | 8 | 0.4475 | 0.4309 | RM257 | 9 | 17666088 | 8 | 0.7014 | 0.6667 |
| RM5639 | 3 | 8170587 | 7 | 0.7067 | 0.6616 | RM3533 | 9 | 17833841 | 9 | 0.8034 | 0.7769 |
| RM218 | 3 | 8375236 | 7 | 0.523 | 0.4935 | RM6570 | 9 | 18577235 | 4 | 0.4789 | 0.4419 |
| RM232 | 3 | 9755759 | 6 | 0.7475 | 0.7084 | OSR28 | 9 | 19788732 | 8 | 0.8228 | 0.7991 |
| RM7 | 3 | 9829641 | 6 | 0.6726 | 0.6147 | RM201 | 9 | 19879785 | 7 | 0.7675 | 0.7312 |
| RM7197 | 3 | 9859760 | 7 | 0.7278 | 0.6921 | RM5384 | 9 | 21766984 | 6 | 0.7697 | 0.7324 |
| RM7345 | 3 | 10958945 | 6 | 0.5825 | 0.5367 | RM1013 | 9 | 22509929 | 6 | 0.7162 | 0.6651 |
| RM282 | 3 | 12408774 | 10 | 0.837 | 0.8173 | RM7492 | 10 | 33968 | 10 | 0.811 | 0.7925 |
| RM338 | 3 | 13222928 | 5 | 0.6261 | 0.5701 | RM244 | 10 | 3612888 | 6 | 0.2903 | 0.2755 |
| RM7403 | 3 | 16668932 | 2 | 0.2311 | 0.2044 | RM7545 | 10 | 3785115 | 20 | 0.9152 | 0.9091 |
| RM16 | 3 | 23127725 | 4 | 0.3682 | 0.3288 | RM216 | 10 | 4986973 | 3 | 0.542 | 0.4789 |
| RM6266 | 3 | 23624397 | 4 | 0.6134 | 0.5497 | RM6646 | 10 | 5361629 | 6 | 0.7317 | 0.6918 |
| RM3513 | 3 | 25114729 | 3 | 0.2203 | 0.2007 | RM311 | 10 | 9818763 | 7 | 0.8402 | 0.8194 |
| RM7097 | 3 | 26680994 | 7 | 0.7139 | 0.6745 | RM184 | 10 | 16430316 | 4 | 0.5398 | 0.4601 |
| RM135 | 3 | 27218612 | 7 | 0.7062 | 0.6685 | RM1125 | 10 | 17327820 | 10 | 0.7816 | 0.7556 |
| RM168 | 3 | 27898585 | 7 | 0.4721 | 0.4444 | RM258 | 10 | 17570591 | 5 | 0.641 | 0.5675 |
| RM186 | 3 | 28813642 | 6 | 0.7365 | 0.6965 | RM269 | 10 | 18026889 | 4 | 0.5092 | 0.4608 |
| RM183 | 3 | 29313123 | 8 | 0.83 | 0.8076 | RM304 | 10 | 18211874 | 2 | 0.0452 | 0.0442 |
| RM5475 | 3 | 30376088 | 12 | 0.8729 | 0.86 | RM5629 | 10 | 18230457 | 8 | 0.7891 | 0.7588 |
| RM416 | 3 | 31255718 | 4 | 0.6171 | 0.5378 | RM6100 | 10 | 18372167 | 4 | 0.3281 | 0.3029 |
| RM448 | 3 | 31399585 | 10 | 0.8514 | 0.8351 | RM1108 | 10 | 18716363 | 3 | 0.0665 | 0.065 |
| RM6712 | 3 | 35020004 | 9 | 0.7809 | 0.7538 | RM171 | 10 | 19120546 | 2 | 0.32 | 0.2688 |
| RM148 | 3 | 35629247 | 5 | 0.6676 | 0.5996 | RM3773 | 10 | 19447567 | 10 | 0.8475 | 0.8297 |
| RM335 | 4 | 679924 | 12 | 0.7687 | 0.7441 | RM5352 | 10 | 20672962 | 4 | 0.4402 | 0.379 |
| RM518 | 4 | 2021760 | 8 | 0.7331 | 0.6913 | RM333 | 10 | 21924163 | 4 | 0.2125 | 0.2022 |
| RM3471 | 4 | 6279483 | 15 | 0.9041 | 0.8961 | RM6160 | 10 | 22048086 | 5 | 0.3647 | 0.3416 |
| RM307 | 4 | 13151235 | 7 | 0.7968 | 0.7686 | RM590 | 10 | 22594389 | 7 | 0.7179 | 0.6796 |
| RM5687 | 4 | 15927814 | 9 | 0.4921 | 0.4773 | RM6327 | 11 | 364257 | 11 | 0.8392 | 0.8199 |
| RM6314 | 4 | 18627879 | 10 | 0.7198 | 0.6805 | RM286 | 11 | 383875 | 6 | 0.5602 | 0.5177 |
| RM471 | 4 | 19007714 | 7 | 0.7105 | 0.6737 | RM1240 | 11 | 1464758 | 12 | 0.8019 | 0.7805 |
| RM4835 | 4 | 19135687 | 5 | 0.5608 | 0.4738 | RM7557 | 11 | 2327475 | 4 | 0.3886 | 0.3539 |
| RM5951 | 4 | 19965149 | 4 | 0.5793 | 0.5087 | RM1812 | 11 | 2392086 | 5 | 0.3595 | 0.3131 |
| RM142 | 4 | 20690898 | 9 | 0.7555 | 0.7218 | RM6544 | 11 | 3838197 | 2 | 0.307 | 0.2599 |
| RM6997 | 4 | 21281309 | 9 | 0.7623 | 0.7344 | RM167 | 11 | 4057566 | 4 | 0.4916 | 0.436 |
| RM7563 | 4 | 22848896 | 8 | 0.841 | 0.8203 | RM3133 | 11 | 6109517 | 5 | 0.6847 | 0.6317 |
| RM6589 | 4 | 27477997 | 6 | 0.7535 | 0.7168 | RM3701 | 11 | 8024726 | 8 | 0.704 | 0.6691 |
| RM317 | 4 | 29219406 | 6 | 0.6717 | 0.6291 | RM7391 | 11 | 9840093 | 7 | 0.7729 | 0.74 |
| RM6089 | 4 | 29563534 | 8 | 0.7288 | 0.7011 | RM7120 | 11 | 11685002 | 5 | 0.5955 | 0.5318 |
| RM3836 | 4 | 31845534 | 9 | 0.7981 | 0.7736 | RM7303 | 11 | 16396823 | 4 | 0.0989 | 0.0969 |
| RM349 | 4 | 32718532 | 8 | 0.67 | 0.6387 | RM287 | 11 | 16610716 | 8 | 0.7714 | 0.7407 |
| RM348 | 4 | 32869438 | 3 | 0.3222 | 0.2739 | RM209 | 11 | 18274583 | 3 | 0.3728 | 0.3292 |
| RM280 | 4 | 35209912 | 3 | 0.1685 | 0.1618 | RM457 | 11 | 18864049 | 4 | 0.3303 | 0.2923 |
| RM559 | 4 | 35371933 | 5 | 0.6635 | 0.602 | RM5349 | 11 | 18985185 | 4 | 0.6005 | 0.5434 |
| RM153 | 5 | 158460 | 10 | 0.8085 | 0.787 | RM21 | 11 | 19639219 | 9 | 0.7278 | 0.6862 |
| RM1182 | 5 | 279748 | 8 | 0.8103 | 0.783 | RM206 | 11 | 21626840 | 13 | 0.8431 | 0.8285 |
| RM122 | 5 | 279748 | 6 | 0.1401 | 0.1376 | RM7170 | 11 | 24420094 | 8 | 0.8162 | 0.7905 |
| RM159 | 5 | 456890 | 12 | 0.8767 | 0.8654 | RM7163 | 11 | 26796502 | 2 | 0.0863 | 0.0826 |
| RM267 | 5 | 2821024 | 7 | 0.7919 | 0.7663 | RM224 | 11 | 26796502 | 12 | 0.8484 | 0.8317 |
| RM405 | 5 | 3073430 | 6 | 0.6461 | 0.6034 | RM6293 | 11 | 28157023 | 6 | 0.6313 | 0.5603 |
| RM574 | 5 | 3390564 | 5 | 0.7756 | 0.738 | RM20 | 12 | 971495 | 10 | 0.6976 | 0.6463 |
| RM437 | 5 | 3815948 | 2 | 0.3735 | 0.3038 | RM19 | 12 | 2433078 | 10 | 0.8055 | 0.7793 |
| RM3193 | 5 | 4965384 | 5 | 0.7481 | 0.7043 | RM247 | 12 | 3185544 | 9 | 0.7988 | 0.7701 |
| RM6082 | 5 | 8799566 | 9 | 0.817 | 0.7932 | RM6296 | 12 | 3200705 | 3 | 0.3662 | 0.3244 |
| RM249 | 5 | 10676235 | 7 | 0.6909 | 0.6338 | RM7619 | 12 | 4828887 | 3 | 0.4705 | 0.3945 |
| RM598 | 5 | 16676126 | 5 | 0.5302 | 0.489 | RM5746 | 12 | 5092032 | 12 | 0.8564 | 0.8413 |
| RM164 | 5 | 19114842 | 5 | 0.604 | 0.5665 | RM512 | 12 | 5104402 | 3 | 0.0873 | 0.0853 |
| RM161 | 5 | 20902803 | 14 | 0.8579 | 0.8425 | RM1337 | 12 | 12042418 | 4 | 0.5615 | 0.4738 |
| RM305 | 5 | 21007006 | 7 | 0.609 | 0.5329 | RM7102 | 12 | 13258404 | 7 | 0.7518 | 0.7238 |
| RM188 | 5 | 22733956 | 9 | 0.8382 | 0.8188 | RM511 | 12 | 17442508 | 5 | 0.7459 | 0.7073 |
| RM480 | 5 | 27155688 | 8 | 0.7053 | 0.6729 | RM277 | 12 | 18319119 | 3 | 0.3512 | 0.2935 |
| RM3170 | 5 | 27796435 | 9 | 0.7939 | 0.7643 | RM1246 | 12 | 19156149 | 7 | 0.6886 | 0.6497 |
| RM5818 | 5 | 29529228 | 8 | 0.7385 | 0.7054 | RM309 | 12 | 21521910 | 7 | 0.7542 | 0.717 |
| RM508 | 6 | 435648 | 8 | 0.7899 | 0.7634 | RM463 | 12 | 22159508 | 4 | 0.4341 | 0.393 |
| RM8109 | 6 | 486929 | 9 | 0.742 | 0.6985 | RM6869 | 12 | 22286876 | 10 | 0.7943 | 0.7732 |
| RM510 | 6 | 2831543 | 9 | 0.7489 | 0.7201 | RM3331 | 12 | 23528087 | 6 | 0.5042 | 0.471 |
| RM225 | 6 | 3416638 | 11 | 0.7649 | 0.7407 | RM5479 | 12 | 24446205 | 13 | 0.7533 | 0.7301 |
| RM314 | 6 | 4845258 | 8 | 0.8037 | 0.7756 | RM270 | 12 | 25002547 | 6 | 0.7285 | 0.6793 |
| RM2126 | 6 | 5917121 | 7 | 0.7696 | 0.7314 | RM17 | 12 | 26988415 | 9 | 0.8183 | 0.7947 |
| RM276 | 6 | 6241911 | 8 | 0.7989 | 0.771 | RM12 | 12 | 27024527 | 4 | 0.4784 | 0.4076 |
| RM50 | 6 | 6376948 | 7 | 0.6467 | 0.6109 |  |  |  |  |  |  |
| Total Alleles | |  |  |  |  |  |  |  | 1948 |  |  |
| Mean | |  |  |  |  |  |  |  | 7.46 | 0.6734 | 0.6395 |

^a^ PIC, Polymorphism information content
