# Supplementary figures and images for: Identification of putative markers linked to grain plumpness in rice (Oryza sativa L.) via association mapping
Source: BMC Genet. 2017 Oct 12;18:89. doi: 10.1186/s12863-017-0559-6 (PMC5639755; doi:10.1186/s12863-017-0559-6)

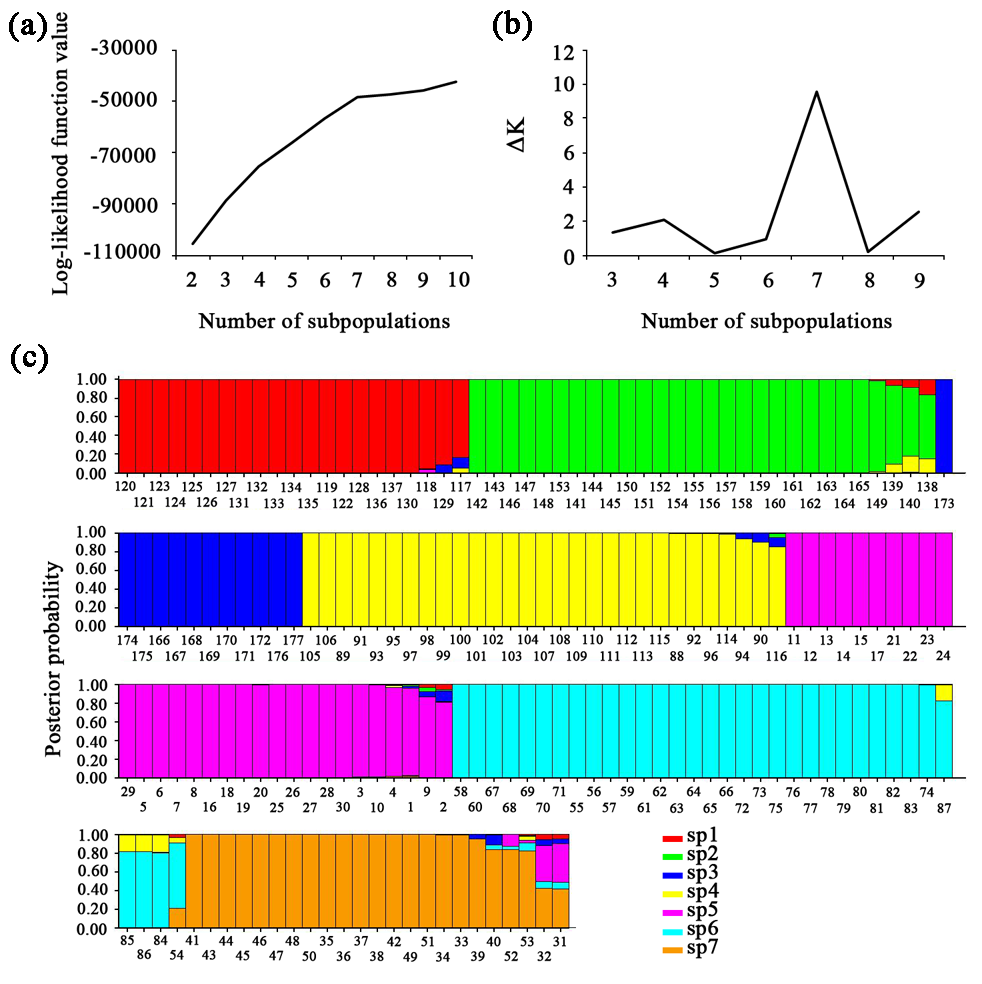

Supplement: Supplementary file 3 — Population genetic architecture analysis of 177 varieties. Effects of changes in log-likelihood function value (a) and the ΔK value (b) on the number of subpopulations and the posterior probabilities of 177 varieties belonging to seven subpopulations (c). Each variety is represented by a vertical bar. The coloured subsections within each vertical bar indicate the membership coefficients (Q) of each variety in different subpopulations. The identified subpopulations are sp1 (red), sp2 (green), sp3 (navy blue), sp4 (yellow), sp5 (purple), sp6 (light blue), and sp7 (brown). (TIFF 291 kb) [file 12863_2017_559_MOESM3_ESM.tif]

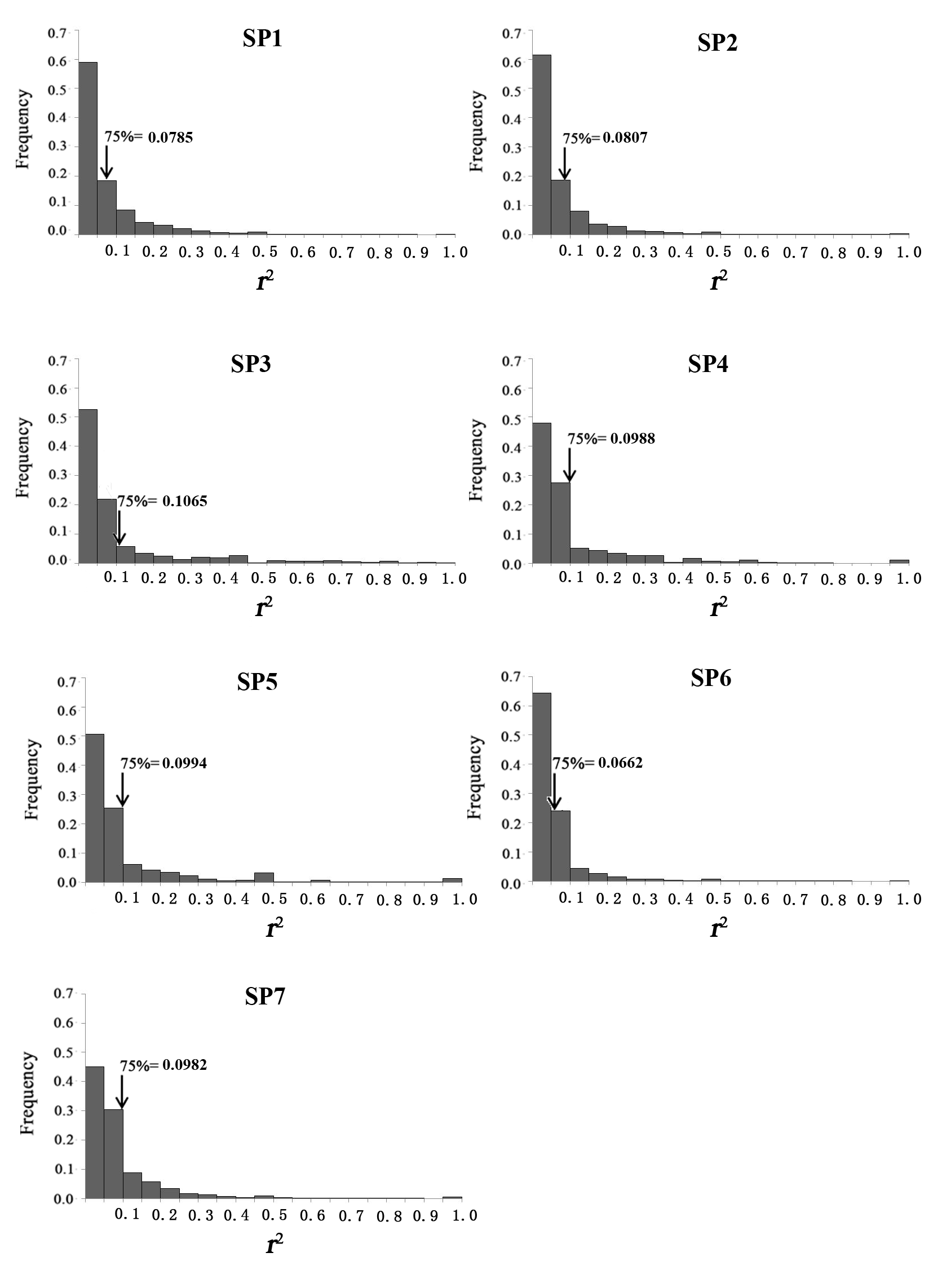

Supplement: Supplementary file 4 — Distribution of the linkage disequilibrium r 2 values between the unlinked SSRs for the seven subpopulations. The 75th percentiles of the r 2 values for the seven subpopulations are indicated. (TIFF 287 kb) [file 12863_2017_559_MOESM4_ESM.tif]

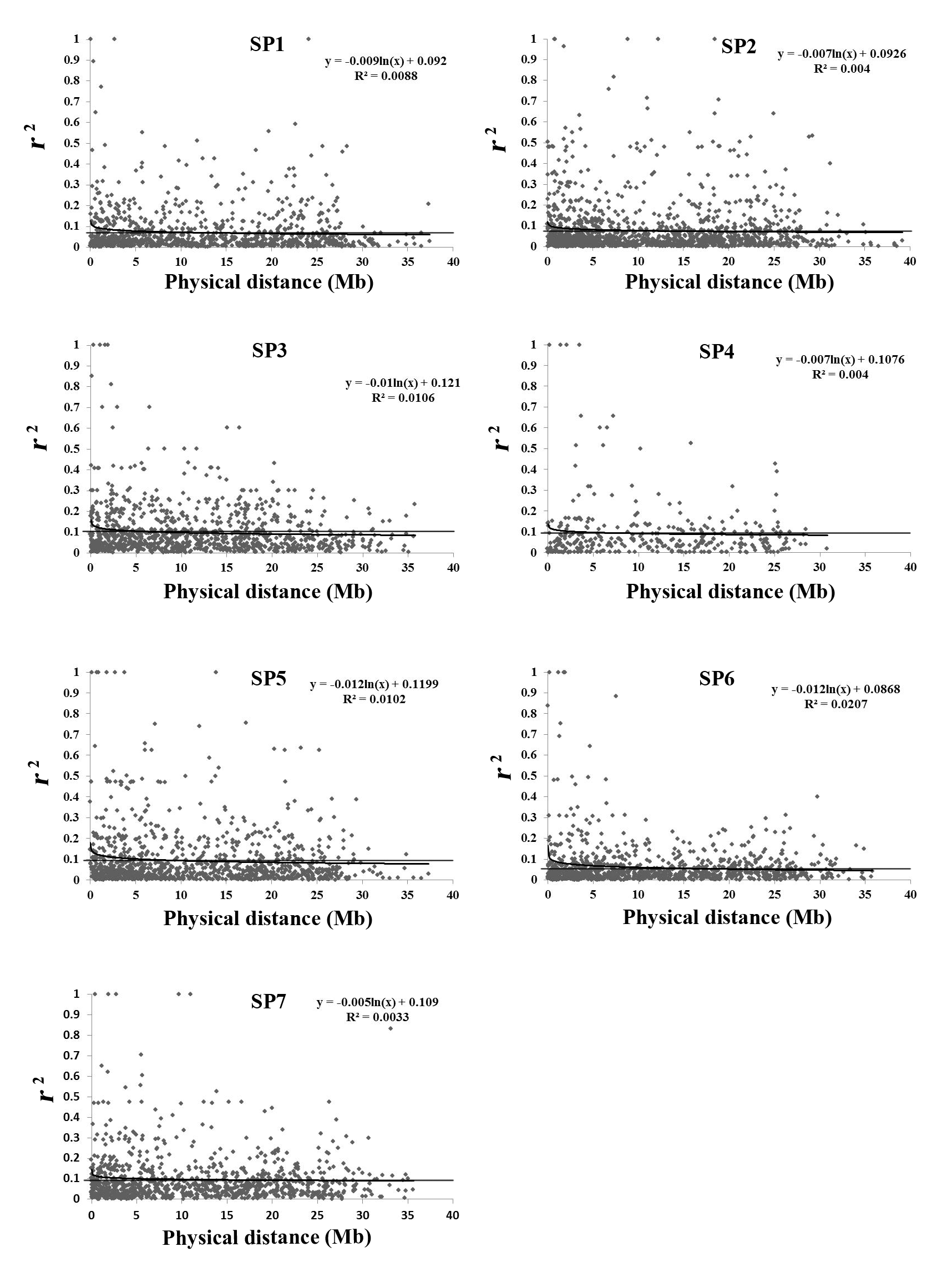

Supplement: Supplementary file 5 — Relationship between the r 2 value and genetic distance for the linked SSR marker pairs for the seven subpopulations. The horizontal line indicates the 75th percentile determined for the distribution of the unlinked SSRs. (TIFF 766 kb) [file 12863_2017_559_MOESM5_ESM.tif]
