# Supplementary material for: Identification of putative markers linked to grain plumpness in rice (Oryza sativa L.) via association mapping
Source: BMC Genet. 2017 Oct 12;18:89. doi: 10.1186/s12863-017-0559-6 (PMC5639755; doi:10.1186/s12863-017-0559-6)
Supplement: Supplementary file 6 — Marker-trait association loci with P < 0.01 determined by the GLM and MLM analyses, their FDRs, proportions of phenotypic variance explained, AAE values, and marker positions on the chromosome derived for 261 markers and 160 rice varieties. (DOCX 27 kb) [file 12863_2017_559_MOESM6_ESM.docx]

**Table S3.** Marker-trait association loci with *P* < 0.01 determined by the GLM and MLM analyses, their FDRs, proportions of phenotypic variance explained, AAE values, and marker positions on the chromosome derived for 261 markers and 160 rice varieties

| Marker | Start position (bp) | Method/location/year | PVE^a^ (%) | *P*-value | FDR^b^ | AAE^c^ (%) | |
| --- | --- | --- | --- | --- | --- | --- | --- |
|  |  |  |  |  |  | Positive | Negative |
| RM8095 | 11238348(1) | GLM/JEF^d^/2013 | 10.75 | 1.60E-03 | 3.57E-03 | 0.80 | -0.35 |
|  |  | GLM/JEF/2014 | 10.22 | 2.00E-03 | 4.19E-03 |  |  |
| RM5 | 23968577(1) | GLM/XF^e^/2013 | 12.55 | 4.50E-03 | 6.25E-03 | 0.70 | -1.02 |
|  |  | GLM/XF/2014 | 12.61 | 4.50E-03 | 4.29E-03 |  |  |
| RM14 | 41363934(1) | GLM/XF/2013 | 13.69 | 1.40E-03 | 2.50E-03 | 2.08 | -1.43 |
|  |  | GLM/JEF/2013 | 12.73 | 2.20E-03 | 4.29E-03 |  |  |
|  |  | GLM/JEF/2014 | 11.83 | 3.30E-03 | 5.81E-03 |  |  |
| **RM5340** | 7482346(2) | GLM/YF^f^/2013 | 16.07 | 1.80E-03 | 1.67E-03 | 1.59 | -1.42 |
|  |  | GLM/YF/2014 | 17.32 | 6.71E-04 | 8.33E-04 |  |  |
|  |  | **MLM/YF/2013** | **15.83** | **7.97E-03** | **1.00E-02** |  |  |
|  |  | **MLM/YF/2014** | **18.26** | **2.39E-03** | **1.00E-02** |  |  |
| RM300 | 13190528(2) | GLM/JEF/2013 | 13.60 | 9.86E-04 | 2.86E-03 | 1.25 | -0.83 |
|  |  | GLM/JEF/2014 | 15.15 | 1.91E-04 | 1.29E-03 |  |  |
| RM573 | 27965302(2) | GLM/JEF/2013 | 17.62 | 3.50E-03 | 5.36E-03 | 1.59 | -0.73 |
|  |  | GLM/JEF/2014 | 17.86 | 2.10E-03 | 4.52E-03 |  |  |
| RM263 | 25889828(2) | GLM/JEF/2013 | 14.39 | 8.10E-03 | 9.29E-03 | 0.83 | -1.04 |
|  |  | GLM/JEF/2014 | 14.11 | 6.90E-03 | 7.42E-03 |  |  |
| RM450 | 28652656(2) | GLM/XF/2013 | 9.63 | 4.70E-03 | 6.88E-03 | 1.89 | -1.07 |
|  |  | GLM/JEF/2013 | 9.01 | 6.60E-03 | 8.57E-03 |  |  |
|  |  | GLM/JEF/2014 | 10.25 | 1.90E-03 | 3.55E-03 |  |  |
| **RM5480** | 5306122(3) | GLM/XF/2013 | 7.24 | 7.70E-03 | 8.75E-03 | 1.56 | -2.05 |
|  |  | GLM/JEF/2013 | 9.37 | 1.30E-03 | 3.21E-03 |  |  |
|  |  | GLM/JEF/2014 | 8.64 | 1.90E-03 | 3.87E-03 |  |  |
|  |  | **MLM/JEF/2013** | **9.38** | **4.27E-03** | **5.00E-03** |  |  |
|  |  | **MLM/JEF/2014** | **9.04** | **4.93E-03** | **6.67E-03** |  |  |
| **RM148** | 35629247(3) | GLM/XF/2013 | 10.55 | 9.73E-04 | 1.88E-03 | 1.32 | -0.28 |
|  |  | GLM/XF/2014 | 9.30 | 2.90E-03 | 2.86E-03 |  |  |
|  |  | **MLM/XF/2013** | **10.69** | **2.76E-03** | **7.50E-03** |  |  |
|  |  | **MLM/XF/2014** | **9.63** | **5.44E-03** | **6.67E-03** |  |  |
| RM7563 | 22848896(4) | GLM/JEF/2013 | 15.50 | 2.75E-04 | 1.43E-03 | 1.26 | -1.28 |
|  |  | GLM/JEF/2014 | 15.61 | 1.64E-04 | 9.68E-04 |  |  |
| RM153 | 158460(5) | GLM/JEF/2013 | 13.86 | 5.70E-03 | 6.79E-03 | 2.35 | -0.88 |
|  |  | GLM/YF/2014 | 14.30 | 5.90E-03 | 5.83E-03 |  |  |
| RM159 | 456890(5) | GLM/JEF/2013 | 19.38 | 2.91E-04 | 1.79E-03 | 1.29 | -1.32 |
|  |  | GLM/JEF/2014 | 18.37 | 4.05E-04 | 2.26E-03 |  |  |
| RM3193 | 4965384(5) | GLM/JEF/2013 | 8.11 | 6.20E-03 | 7.14E-03 | 0.39 | -1.30 |
|  |  | GLM/JEF/2014 | 7.36 | 9.40E-03 | 9.36E-03 |  |  |
| RM8109 | 486929(6) | GLM/JEF/2013 | 11.91 | 6.40E-03 | 7.86E-03 | 0.39 | -0.54 |
|  |  | GLM/JEF/2014 | 13.15 | 2.10E-03 | 4.84E-03 |  |  |
| RM510 | 2831543(6) | GLM/JEF/2013 | 15.44 | 4.79E-04 | 2.14E-03 | 1.50 | -1.38 |
|  |  | GLM/JEF/2014 | 13.46 | 1.70E-03 | 2.90E-03 |  |  |
| RM2530 | 15570383(7) | GLM/YF/2013 | 14.04 | 4.30E-03 | 6.67E-03 | 1.11 | -0.96 |
|  |  | GLM/YF/2014 | 16.31 | 8.28E-04 | 1.67E-03 |  |  |
|  |  | GLM/XF/2013 | 15.85 | 7.80E-04 | 1.25E-03 |  |  |
|  |  | GLM/XF/2014 | 12.99 | 6.70E-03 | 8.57E-03 |  |  |
|  |  | GLM/JEF/2013 | 18.21 | 9.50E-05 | 1.07E-03 |  |  |
|  |  | GLM/JEF/2014 | 16.85 | 1.91E-04 | 1.61E-03 |  |  |
| **RM505** | 24527931(7) | GLM/XF/2013 | 18.78 | 4.32E-06 | 6.25E-04 | 1.68 | -1.72 |
|  |  | GLM/XF/2014 | 15.47 | 8.91E-05 | 1.43E-03 |  |  |
|  |  | **MLM/XF/2013** | **14.02** | **1.47E-03** | **2.50E-03** |  |  |
|  |  | **MLM/XF/2014** | **12.69** | **2.52E-03** | **3.33E-03** |  |  |
| RM3589 | 25054610(7) | GLM/JEF/2013 | 14.53 | 3.40E-03 | 5.00E-03 | 1.24 | -0.91 |
|  |  | GLM/JEF/2014 | 15.00 | 1.70E-03 | 3.23E-03 |  |  |
| RM506 | 126285(8) | GLM/YF/2013 | 16.19 | 3.20E-03 | 3.33E-03 | 1.35 | -1.27 |
|  |  | GLM/YF/2014 | 15.81 | 3.20E-03 | 4.17E-03 |  |  |
| **RM1235** | 1203431(8) | GLM/JEF/2013 | 8.88 | 3.10E-03 | 4.64E-03 | 1.26 | -1.48 |
|  |  | GLM/YF/2014 | 9.00 | 3.90E-03 | 5.00E-03 |  |  |
|  |  | GLM/XF/2014 | 8.40 | 5.30E-03 | 5.71E-03 |  |  |
|  |  | **MLM/JEF/2013** | **9.72** | **5.09E-03** | **1.00E-02** |  |  |
|  |  | **MLM/XF/2014** | **9.01** | **7.96E-03** | **1.00E-02** |  |  |
| RM6863 | 2005990(8) | GLM/XF/2013 | 13.36 | 5.40E-03 | 7.50E-03 | 1.98 | -1.18 |
|  |  | GLM/JEF/2013 | 12.61 | 8.00E-03 | 8.93E-03 |  |  |
|  |  | GLM/JEF/2014 | 12.95 | 4.80E-03 | 6.45E-03 |  |  |
| RM544 | 5102982(8) | GLM/XF/2013 | 12.12 | 9.10E-03 | 1.00E-02 | 1.23 | -1.18 |
|  |  | GLM/JEF/2013 | 11.85 | 8.70E-03 | 1.00E-02 |  |  |
| RM331 | 12288130(8) | GLM/YF/2014 | 11.26 | 1.50E-03 | 3.33E-03 | 1.31 | -0.67 |
|  |  | GLM/JEF/2013 | 10.78 | 1.70E-03 | 3.93E-03 |  |  |
|  |  | GLM/JEF/2014 | 9.86 | 2.80E-03 | 5.16E-03 |  |  |
| RM8206 | 5866359(9) | GLM/XF/2013 | 11.63 | 7.80E-03 | 9.38E-03 | 0.47 | -1.24 |
|  |  | GLM/XF/2014 | 12.30 | 5.40E-03 | 7.14E-03 |  |  |
| RM201 | 19879785(9) | GLM/JEF/2013 | 12.49 | 9.61E-04 | 2.50E-03 | 1.42 | -1.1 |
|  |  | GLM/JEF/2014 | 13.52 | 2.96E-04 | 1.94E-03 |  |  |
| RM5629 | 18230457(10) | GLM/JEF/2013 | 11.07 | 3.80E-03 | 6.07E-03 | 1.33 | -1.24 |
|  |  | GLM/JEF/2014 | 10.04 | 6.40E-03 | 7.10E-03 |  |  |
| RM590 | 22594389(10) | GLM/JEF/2013 | 9.09 | 6.30E-03 | 7.50E-03 | 1.06 | -1.58 |
|  |  | GLM/JEF/2014 | 8.32 | 9.40E-03 | 9.68E-03 |  |  |
| **RM511** | 17442508(12) | GLM/JEF/2013 | 13.25 | 7.49E-05 | 7.14E-04 | 1.34 | -1.19 |
|  |  | GLM/JEF/2014 | 13.05 | 6.32E-05 | 6.45E-04 |  |  |
|  |  | **MLM/JEF/2013** | **9.95** | **4.46E-03** | **7.50E-03** |  |  |
|  |  | **MLM/JEF/2014** | **9.61** | **5.52E-03** | **1.00E-02** |  |  |
| RM1246 | 19156149(12) | GLM/YF/2013 | 9.00 | 9.20E-03 | 1.00E-02 | 0.48 | -0.99 |
|  |  | GLM/YF/2014 | 9.05 | 8.50E-03 | 9.17E-03 |  |  |
| **RM5479** | 24446205(12) | GLM/JEF/2013 | 22.28 | 4.59E-06 | 3.57E-04 | 1.67 | -1.18 |
|  |  | GLM/JEF/2014 | 20.64 | 1.11E-05 | 3.23E-04 |  |  |
|  |  | **MLM/JEF/2013** | **21.06** | **7.33E-04** | **2.50E-03** |  |  |
|  |  | **MLM/JEF/2014** | **20.39** | **1.03E-03** | **3.33E-03** |  |  |

^a^ PVE, Percentage of phenotypic variation explained; ^b^ FDR, False discovery rate; ^c^ AAE, Average allele effect; ^d^ JEF, Jiangpu Experimental Farm; ^e^ XF, Xinyang Farm; ^f^ YF, Yuanyang Farm; Bold markers represent that they were also detected by the MLM analyses; Digit in parentheses of the second column is the chromosome number
