# Supplementary material for: Identification of putative markers linked to grain plumpness in rice (Oryza sativa L.) via association mapping
Source: BMC Genet. 2017 Oct 12;18:89. doi: 10.1186/s12863-017-0559-6 (PMC5639755; doi:10.1186/s12863-017-0559-6)
Supplement: Supplementary file 7 — Alleles at seven maker-trait loci in typical carrier varieties. ‘√’ indicates that the variety has the positive allele, and ‘∆’ indicates that the variety has the negative allele. (DOCX 22 kb) [file 12863_2017_559_MOESM7_ESM.docx]

**Table S4. Alleles at seven maker-trait loci in typical carrier varieties**

| Locus-  allele | Phenotypic  effect value (%) | Typical carrier variety | | | | |
| --- | --- | --- | --- | --- | --- | --- |
|  |  | Yazihuang | Ligengqing | Huangsanshi | Qiutiandaxiedao | Maozitou |
| RM5340-95 | 0.47 |  |  |  |  | √ |
| RM5340-155 | 1.86 | √ | √ |  |  |  |
| RM5340-205 | 2.44 |  |  |  |  |  |
| RM5340-145 | -0.21 |  |  |  |  |  |
| RM5340-150 | -0.29 |  |  |  |  |  |
| RM5340-160 | -0.47 |  |  | Δ | Δ |  |
| RM5340-165 | -2.62 |  |  |  |  |  |
| RM5480-165 | 1.79 |  |  |  |  |  |
| RM5480-200 | 1.32 |  | √ | √ |  |  |
| RM5480-180 | -0.54 |  |  |  | Δ |  |
| RM5480-190 | -3.56 | Δ |  |  |  |  |
| RM148-125 | 1.32 |  | √ | √ |  |  |
| RM148-130 | -0.14 |  |  |  |  | Δ |
| RM148-135 | -0.42 |  |  |  | Δ |  |
| RM505-160 | 1.96 |  |  |  | √ |  |
| RM505-170 | 2.62 |  |  |  |  | √ |
| RM505-180 | 0.47 | √ |  |  |  |  |
| RM505-175 | -2.86 |  |  |  |  |  |
| RM505-190 | -1.91 |  |  |  |  |  |
| RM505-195 | -0.38 |  | Δ | Δ |  |  |
| RM1235-120 | 1.26 | √ | √ | √ |  | √ |
| RM1235-105 | -1.48 |  |  |  |  |  |
| RM1235-110 | -0.67 |  |  |  | Δ |  |
| RM511-130 | 2.39 |  | √ | √ |  |  |
| RM511-135 | 0.28 | √ |  |  |  | √ |
| RM511-125 | -1.15 |  |  |  | Δ |  |
| RM511-140 | -1.81 |  |  |  |  |  |
| RM511-150 | -0.61 |  |  |  |  |  |
| RM5479-210 | 1.83 |  | √ | √ |  |  |
| RM5479-215 | 2.48 |  |  |  |  |  |
| RM5479-225 | 0.70 |  |  |  | √ |  |
| RM5479-185 | -2.20 |  |  |  |  |  |
| RM5479-200 | -1.15 |  |  |  |  |  |
| RM5479-205 | -0.20 |  |  |  |  |  |

‘√’ indicates that the variety has the positive allele, and ‘∆’ indicates that the variety has the negative allele

**Supplementary Table 3** (Continued)

| Locus-  allele | Phenotypic  effect value (%) | Typical carrier variety | | | | |
| --- | --- | --- | --- | --- | --- | --- |
|  |  | Chiguhong | Qiaobinghuang | Zaoguangtou | Yuedao 5 | Yuedao 22 |
| RM5340-95 | 0.47 |  |  |  | √ |  |
| RM5340-155 | 1.86 |  |  |  |  |  |
| RM5340-205 | 2.44 |  |  |  |  | √ |
| RM5340-145 | -0.21 | Δ |  |  |  |  |
| RM5340-150 | -0.29 |  |  |  |  |  |
| RM5340-160 | -0.47 |  | Δ | Δ |  |  |
| RM5340-165 | -2.62 |  |  |  |  |  |
| RM5480-165 | 1.79 |  |  |  | √ | √ |
| RM5480-200 | 1.32 |  |  | √ |  |  |
| RM5480-180 | -0.54 | Δ |  |  |  |  |
| RM5480-190 | -3.56 |  | Δ |  |  |  |
| RM148-125 | 1.32 |  | √ | √ |  | √ |
| RM148-130 | -0.14 | Δ |  |  | Δ |  |
| RM148-135 | -0.42 |  |  |  |  |  |
| RM505-160 | 1.96 |  |  |  |  |  |
| RM505-170 | 2.62 |  |  |  | √ |  |
| RM505-180 | 0.47 | √ |  |  |  |  |
| RM505-175 | -2.86 |  |  |  |  |  |
| RM505-190 | -1.91 |  |  |  |  |  |
| RM505-195 | -0.38 |  | Δ | Δ |  |  |
| RM1235-120 | 1.26 |  | √ | √ | √ | √ |
| RM1235-105 | -1.48 |  |  |  |  |  |
| RM1235-110 | -0.67 | Δ |  |  |  |  |
| RM511-130 | 2.39 |  | √ | √ |  | √ |
| RM511-135 | 0.28 |  |  |  | √ |  |
| RM511-125 | -1.15 | Δ |  |  |  |  |
| RM511-140 | -1.81 |  |  |  |  |  |
| RM511-150 | -0.61 |  |  |  |  |  |
| RM5479-210 | 1.83 |  |  |  |  |  |
| RM5479-215 | 2.48 |  |  | √ |  |  |
| RM5479-225 | 0.70 |  |  |  | √ | √ |
| RM5479-185 | -2.20 |  |  |  |  |  |
| RM5479-200 | -1.15 | Δ |  |  |  |  |
| RM5479-205 | -0.20 |  |  |  |  |  |

‘√’ indicates that the variety has the positive allele, and ‘∆’ indicates that the variety has the negative allele
