# Supplementary material for: Identification of putative markers linked to grain plumpness in rice (Oryza sativa L.) via association mapping
Source: BMC Genet. 2017 Oct 12;18:89. doi: 10.1186/s12863-017-0559-6 (PMC5639755; doi:10.1186/s12863-017-0559-6)
Supplement: Supplementary file 8 — Comparison of SSR markers identified in this study and genes or QTLs reported in previous studies. The physical positions of the third and fifth columns were determined based on data from Gramene (http://www.gramene.org/markers) and NCBI (http://blast.ncbinlm.nih.gov/Blast.cgi). (DOCX 19 kb) [file 12863_2017_559_MOESM8_ESM.docx]

**Table S5. Comparison of SSR markers identified in this study and genes or QTLs reported in previous studies**

| Marker | Chromosome | Start position (bp)^a^ | Genes or QTLs reported in the previous studies | | | Difference (bp) |
| --- | --- | --- | --- | --- | --- | --- |
|  |  |  | Symbol | Position (bp)^a^ | Reference |  |
| RM5340 | 2 | 7,482,346 | qGR-2-1 | 595,109-4,305,907 | Jia et al., 2012 | 3,176,439 |
|  |  |  | GW2 | 8,114,961-8,121,925 | Song et al., 2007 | 632,615 |
| RM5480 | 3 | 5,306,122 | OsMADS1 | 6,053,188-6,061,274 | Prasad et al., 2005 | 747,066 |
| RM148 | 3 | 35,629,247 | TGW3b | 30,308,684-30,570,344 | Liu et al., 2010 | 5,320,563 |
| RM505 | 7 | 24,527,931 | qGR-7-7 | 2,678,661-21,045,102 | Jia et al., 2012 | 3,482,829 |
|  |  |  | qGR-7-8 | 21,045,102-26,636,625 | Jia et al., 2012 | in |
|  |  |  | SUS3 | 25,429,639-25,435,132 | Hirose et al., 2008 | 901,708 |
|  |  |  | GL7 | 24,664,168-24,669,324 | Wang et al., 2015 | 136,237 |
|  |  |  | GE | 24,713,778-24,715,813 | Nagasawa et al., 2013 | 185,847 |
|  |  |  | qGP-7 | 28,411,532-29,466,858 | Niu et al., 2004 | 3,883,601 |
| RM1235 | 8 | 1,203,431 | DTH8 | 4,332,106-4,334,829 | Wei et al., 2010 | 3,128,675 |
|  |  |  | RM44 | 11,759,419-11,759,434 | Takai et al., 2005 | 10,555,988 |
| RM511 | 12 | 17,442,508 | RM511 | 17,442,508 | Liu et al., 2015 | 0 |
| RM5479 | 12 | 24,446,205 | C1069 | 25,080,619-25,081,017 | Takai et al., 2005 | 634,414 |
|  |  |  | OsSUT2 | 27,513,939-27,518,251 | Takeda et al., 2001 | 3,067,734 |

^a^The physical positions of the third and fifth columns were determined based on data from Gremene (http://www.gramene.org/markers) and NCBI (http://blast.ncbinlm.nih.gov/Blast.cgi)
